# Does the Mean Platelet Volume Decrease in the Presence of Coronary Artery Fistula?

**DOI:** 10.5935/abc.20190154

**Published:** 2019-08

**Authors:** Henrique Trombini Pinesi, Roberto Rocha C. V. Giraldez

**Affiliations:** Instituto do Coração (InCor) - HCFMUSP, São Paulo, SP - Brazil

**Keywords:** Platelet Activation/genetic, Acute Coronary Syndrome, Mean Platelet Volume, Inflammation, Coronary Angiography

It has long been known that platelet activation is involved in the genesis of several cardiovascular diseases, especially acute coronary syndromes and other atherosclerotic diseases.^1^ Studies carried out in the 1970s already showed that the endothelial lesion was capable of triggering a cascade of inflammatory events leading to platelet activation and consequent vascular thrombosis.^2^

Activated platelets have a larger size due to their increased enzymatic and metabolic activity.^3^ These observations led to a series of studies that evaluated the correlation between mean platelet volume (MVP) and cardiovascular disease. Most of these studies found a positive correlation between these variables, with a higher risk of ischemic events in patients with higher MPV.^4,5^ These studies were replicated in several different situations, with similar results. Despite this, they have never been tested in large clinical trials as part of the decision-making. Therefore, there is no robust evidence to use MPV or even more complex platelet activity tests in daily clinical practice as a cardiovascular risk factor up to the present time.^6,7^

Coronary artery fistulas (CAF) are rare findings, present in approximately 0.2% of adults submitted to coronary angiography.^8^ The main etiology is congenital, with a recent increase in the etiology of acquired CAF due to the increased number of invasive procedures with the development of hemodynamics.^9^

Most of the time, the CAF are small and clinically asymptomatic, not requiring specific treatment. In exceptional cases, when there is drainage to the right chambers and the fistula flow rate is high, the phenomenon of “coronary steal” may occur, with decreased blood flow to the myocardium and local ischemia, especially in situations of increased oxygen demand, such as during physical exertion. In these situations, the patient may have chest pain and need some interventional treatment.^10^ Most fistulas are not correlated with an increased risk of myocardial ischemic events, but early atherosclerosis may occur in case of persistent high-debt fistulae.^11^

The study carried out by Sincer et al.^12^ sought to evaluate the presence of a correlation between MPV and CAF. In the analyzed population, a negative correlation was observed between these factors, with the lower MPV being related to the presence of CAF. This finding differs from that seen in other cardiovascular diseases, in which there is an increase in MPV, as previously mentioned. Since the coronary fistula is not an inflammatory disease and is not correlated with an increased risk of atherosclerotic events, this finding may be real.

The pathophysiological explanation for this finding, however, is unknown and its practical applicability is extremely limited. The observation of the correlation between MPV and CAF may also have been merely a random fact, albeit statistically significant.

This is a common occurrence when one tests the correlation of several variables with one outcome. Further studies involving the analysis of platelet activation in atherosclerotic and non-atherosclerotic coronary diseases are still necessary to add this information to our daily clinical practice, both as a risk marker and, eventually, as therapy-guiding factor.
